# Data on occurrence and fate of emerging contaminants in a urbanised area

**DOI:** 10.1016/j.dib.2018.01.029

**Published:** 2018-01-31

**Authors:** Sara Castiglioni, Enrico Davoli, Francesco Riva, Marinella Palmiotto, Paolo Camporini, Angela Manenti, Ettore Zuccato

**Affiliations:** a IRCCS – Istituto di Ricerche Farmacologiche “Mario Negri”, Department of Environmental Health Sciences, Via La Masa 19, 20156 Milan, Italy; b Metropolitana Milanese S.p.A., Area Acquedotto, Via Giuseppe Meda 44, 20141 Milan, Italy

## Abstract

These data and analyses support the research article “Mass balance of emerging contaminants in the water cycle of an highly urbanized and industrialized area of Italy” by Castiglioni et al. (2018) [1].

The occurrence of 80 emerging contaminats in waste and surface water was investigated in an highly urbanised area of Italy, the River Lambro basin. The data presented here include: (1) concentrations in untreated and treated wastewater of different wastewater treatment plants (WWTPs); (2) concentrations in surface water collected along the river Lambro, in the north and south of the city of Milan (main urban center in the area). These concentrations indicate the distribution and fate of emerging contaminats in the environment.

**Specifications Table**

**Table:** 

Subject area	*Analytical Chemistry*
More specific subject area	*Emerging Contaminants in the environment*
Type of data	*Tables*
How data was acquired	*Mass spectrometry (*API 3000 QqQ*, ABSciex;* 6410 QqQ Agilent Technologies)
Data format	*Raw data*
Experimental factors	*Samples were filtered and extracted by solid phase extraction*
Experimental features	*Samples were collected in the influents and effluents of three wastewater treatment plants in Milan, and in rivers receiving discharges from the plants and the surrounding urbanised area. Wastewater effluents were collected taking into account the wastewater resident time in the plant.*
Data source location	*Milan and River Lambro basin; North of Italy*
Data accessibility	*The data are available within this article.*
Related research article	*This data article is a companion paper of the research article:*
	*Castiglioni, S.; Davoli, E., Riva F., Palmiotto, M. Camporini, P. Manenti, A., Zuccato E. 2018. Mass balance of emerging contaminants in the water compartment of an highly urbanized and industrialized area of Italy. Water Research. 131, 287-298.*

**Value of the Data**

• These data offer a comprehensive overview of the occurrence of a wide panel of emerging contaminats in waste and surface water in a urban area and can be compared with other studies.
• These data may help to understand the distribution and fate of the emerging contaminats in the environment.
• These data may contribute to the need of monitoring data to support future prioritisation exercises and guidelines development by national and international authorities.
• The occurrence and distribution of contaminats may help to identify the sources of contamination in a urban area.

## 1 Data

The presented data were obtained during a comprehensive monitoring study in the most urbanised and industrialized area of Italy. The occurrence of about 80 emerging contaminants was investigated in wastewater (WW) and surface water in the river Lambro basin. The fate of these contaminants during wastewater treatment was assessed by analysing influents and effluents in three wastewater treatment plants (WWTPs) which collect wastes from the entire city of Milan. Data presented include: (1) concentrations of emerging contaminats in influent wastewater collected before any treatment (Table 1, Table 2, Table 3; (2) concentrations in effluent wastewater collected immediately before the discharge in surface water (Table 4, Table 5, Table 6; (3) concentrations in rivers Olona, Seveso and Lambro collected before Milan (O1,S1,L1) and in the Lambro River after discharges from the city of Milan (L2,3,4) and at the closure of the basin (L5) (Table 7, Table 8, Table 9). Refer to [1] for detailed interpretation and discussion.

**Table 1:** Means, medians and ranges of pharmaceuticals (*PHARM*) measured in influent wastewater.

**Influent WW**									
**Concentrations (ng/L)**	**WWTP A**			**WWTP B**			**WWTP C**		
	**Mean**	**Median**	**Range**	**Mean**	**Median**	**Range**	**Mean**	**Median**	**Range**
**Antibiotics**									
Amoxicillin	2.0	1.0	LOQ-6	<LOQ	<LOQ	<LOQ	<LOQ	<LOQ	<LOQ
Ciprofloxacin	655.6	632.7	220–1120	666.7	656.5	492–876	531.9	693.2	114–905
Clarithromycin	1012.0	909.1	715–1510	976.1	976.8	404–1617	892.9	960.8	698–1075
Dehydro-erythromycin	307.8	297.6	170–517	303.8	313.9	43–636	196.4	215.5	136–219
Erythromycin	<LOQ	<LOQ	<LOQ	<LOQ	<LOQ	<LOQ	<LOQ	<LOQ	<LOQ
Lincomycin	29.5	29.7	14–40	28.1	28.3	17–40	13.9	14.3	10.4–17.3
Ofloxacin	487.8	469.3	144–830	682.3	631.9	580–908	467.1	624.3	85–738
Oxytetracycline	<LOQ	<LOQ	<LOQ	<LOQ	<LOQ	<LOQ	<LOQ	<LOQ	<LOQ
Spiramycin	<LOQ	<LOQ	<LOQ	<LOQ	<LOQ	<LOQ	<LOQ	<LOQ	<LOQ
Sulfamethoxazole	170.0	93.9	32–1057	10.5	5.9	11–36.8	<LOQ	<LOQ	<LOQ
Vancomicin	64.9	58.3	LOQ-127	<LOQ	<LOQ	<LOQ	<LOQ	<LOQ	<LOQ

**Anticancer**									
Cyclophosphamide	2.9	2.9	LOQ-5.5	1.8	1.0	LOQ-4.2	4.9	3.6	LOQ-10
Methotrexate	3.5	1.4	0.9–28	<LOQ	<LOQ	<LOQ	3.0	2.3	LOQ-8
Tamoxifen	<LOQ	<LOQ	<LOQ	<LOQ	<LOQ	<LOQ	<LOQ	<LOQ	<LOQ

**Antiinflammatory**									
Diclofenac	798.5	611.9	214–2198	494.5	449.7	333–841	731.2	546.5	426–1794
Ibuprofen	1709.7	1707.0	975–2377	1643.4	1641.8	1149–2114	863.9	873.7	660–1049
Ketoprofen	1209.5	1262.9	708–1924	964.2	917.6	792–1242	859.0	792.4	685–1283
Naproxen	1192.5	1207.7	706–1688	1407.9	1072.6	701–5921	571.1	534.1	517–668
Paracetamol	3095.8	3328.3	1961–3960	2471.4	2349.2	1481–3737	2578.4	2683.3	1661–3099

**Bronchodilator**									
Salbutamol	6.9	6.8	5.1–9.8	12.4	10.5	6.6–23.6	8.3	8.4	7.1–10

**Cardiovascular**									
Atenolol	1519.0	1564.7	1142–1789	1913.7	1910.4	1368–2888	1614.2	1615.3	1448–1745
Enalapril	62.9	65.5	41–86	106.2	75.2	57–324	91.1	92.3	71–114

**CNS drug**									
Carbamazepine	286.3	285.1	184–429	309.2	314.1	248–370	1313.7	275.5	221–3487
Demetyl-diazepam	3.7	3.7	2.9–4.3	4.5	4.7	2.2–6.3	3.7	4.0	2.7–4.5
Diazepam	1.7	1.6	1.0–2.3	1.4	1.2	1.0–2.7	6.3	3.7	3.2–22

**Diuretics**									
Furosemide	544.8	474.9	304–1083	429.5	412.8	165–662	548.7	446.1	279–934
Hydrochlorothiazide	667.6	740.8	341–848	547.5	528.4	116–1001	377.6	369.0	317–523

**Estrogens**									
17-β estradiol	15.8	11.8	LOQ-37	6.1	4.6	LOQ-15	15.6	15.1	12.4–19.5
Estrone	41.2	41.6	25–57	53.4	44.6	20–104	36.3	35.9	34–41
17-α ethynilestradiol	<LOQ	<LOQ	<LOQ	<LOQ	<LOQ	<LOQ	<LOQ	<LOQ	<LOQ

**Gastrointestinal**									
Omeprazole	<LOQ	<LOQ	<LOQ	<LOQ	<LOQ	<LOQ	<LOQ	<LOQ	<LOQ
Ranitidine	115.7	106.4	41–234	117.2	114.8	32–217	112.0	109.9	83–165

**Lipid Regulators**									
Atorvastatine	79.1	65.9	24–153	56.2	52.7	28–98	55.5	49.0	18–108
Bezafibrate	148.1	155.5	70–281	156.3	156.1	91–278	2181.7	1780.5	353–6045
Clofibric acid	<LOQ	<LOQ	<LOQ	<LOQ	<LOQ	<LOQ	<LOQ	<LOQ	<LOQ
Gemfibrozil	263.3	204.4	90–787	215.7	229.2	114–295	155.4	154.5	107–197

**Erectile dysfunction drug**									
Sildenafil	<LOQ	<LOQ	<LOQ	<LOQ	<LOQ	<LOQ	<LOQ	<LOQ	<LOQ

**Table 2:** Means, medians and ranges of illicit drugs (IDs) measured in influent wastewater.

**Influent WW**									
**Concentrations (ng/L)**	**WWTP A**			**WWTP B**			**WWTP C**		
	**Mean**	**Median**	**Range**	**Mean**	**Median**	**Range**	**Mean**	**Median**	**Range**
**Cocaine and metabolites**									
Benzoylecgonine	638.9	631.3	480–880	885.9	867.3	630–1300	660.2	618.6	580–860
Norbenzoylecgonine	22.6	21.9	16–31	29.8	29.5	20–50	22.1	19.5	18–32
Cocaine	262.2	251.4	190–325	346.9	337.6	180–615	242.8	246.2	174–293
Norcocaine	4.1	3.9	2.4–5.8	6.7	6.1	4.0–13.0	4.4	4.2	3.8–5.7
Cocaethylene	6.4	5.5	3.9–10.7	9.2	9.0	5.0–20.0	4.7	4.7	4.0–7.0
Ecgonine methyl ester	157.9	153.0	113–228	255.9	244.8	160–405	115.3	123.4	<LOQ-208
Ecgonine	125.3	122.3	<LOQ-300	192.9	189.8	94–312	97.9	115.3	<LOQ-126
Anhydroecgonine	<LOQ	<LOQ	<LOQ	2.6	0.4	<LOQ-10	<LOQ	<LOQ	<LOQ
Anhydroecg. methylester	<LOQ	<LOQ	<LOQ	<LOQ	<LOQ	<LOQ	<LOQ	<LOQ	<LOQ

**Opioids**									
Morphine	59.1	57.7	34–85	49.8	47.4	34–80	68.5	69.7	49–99
6-acethylmorphine	<LOQ	<LOQ	<LOQ-7.5	<LOQ	<LOQ	<LOQ	<LOQ	<LOQ	<LOQ
Morphine 3β glucuronide	2.5	0.6	<LOQ-7.0	6.4	7.0	<LOQ-19	3.9	2.9	<LOQ-12
Morphine 6β glucuronide	2.2	1.5	<LOQ-5	2.0	1.5	<LOQ-4.2	2.8	1.5	<LOQ-5.1
Oxycodone	8.7	2.3	<LOQ-91	31.6	2.3	<LOQ-412	<LOQ	<LOQ	<LOQ
Hydrocodone	<LOQ	<LOQ	<LOQ	<LOQ	<LOQ	<LOQ	<LOQ	<LOQ	<LOQ
Codeine	94.9	72.4	50–390	105.1	108.8	53–153	76.5	75.7	66–91
6-acethylcodeine	<LOQ	<LOQ	<LOQ	<LOQ	<LOQ	<LOQ	<LOQ	<LOQ	<LOQ
Methadone	9.5	9.4	6.0–14.0	17.3	18.0	14–22	8.4	9.4	3.0–11.0
EDDP	12.8	10.6	7.0–21.0	22.4	22.4	15–33	9.2	10.3	4.0–12.0

**Amphetamines and Ketamine**									
Amphetamine	25.2	21.3	<LOQ-45	<LOQ	<LOQ	<LOQ	<LOQ	<LOQ	<LOQ
Methamphetamine	146.5	141.3	112–210	84.5	64.3	8–240	10.7	9.8	9.0–14.0
MDA	<LOQ	<LOQ	<LOQ	<LOQ	<LOQ	<LOQ	<LOQ	<LOQ	<LOQ
MDMA	13.0	10.2	<LOQ-33	7.0	1.6	<LOQ-31	6.2	1.6	<LOQ-18
MDEA	<LOQ	<LOQ	<LOQ	<LOQ	<LOQ	<LOQ	<LOQ	<LOQ	<LOQ
MBDB	<LOQ	<LOQ	<LOQ	<LOQ	<LOQ	<LOQ	<LOQ	<LOQ	<LOQ
Ketamine	6.8	7.0	4.0–9.5	6.8	6.6	<LOQ-16	3.0	3.7	<LOQ-5.3

**Cannabinoids**									
THC-COOH	67.12	61.07	50–120	64.59	63.15	41–90	91.73	74.17	41–164
OH-THC	<LOQ	<LOQ	<LOQ	<LOQ	<LOQ	<LOQ	<LOQ	<LOQ	<LOQ
THC	6.48	5.64	<LOQ-15	<LOQ	<LOQ	<LOQ	<LOQ	<LOQ	<LOQ

**Table 3:** Means, medians and ranges of the other classes of ECs measured in influent wastewater.

**Influent WW**									
**Concentrations (ng/L)**	**WWTP A**			**WWTP B**			**WWTP C**		
	**Mean**	**Median**	**Range**	**Mean**	**Median**	**Range**	**Mean**	**Median**	**Range**
**Personal Care Products (PCPs)**									
PBSA	185.1	183.0	60–327	387.1	361.7	185–573	309.9	316.9	202–458
Benzophenone-4	404.8	392.4	154–638	548.1	512.4	236–1000	186.9	185.9	99–275
Benzophenone-3	48.2	45.2	20–82	53.6	53.5	33–74	35.8	45.1	<LOQ-58
4-MBC	<LOQ	<LOQ	<LOQ	<LOQ	<LOQ	<LOQ	<LOQ	<LOQ	<LOQ

**Disinfectants (DIS)**									
Triclosan	1195	1144	505–2210	976.1	840.9	645–1705	1405	1609	706–1930
Triclocarban	<LOQ	<LOQ	<LOQ	<LOQ	<LOQ	<LOQ	<LOQ	<LOQ	<LOQ

**Perfluorinated compounds (PERF)**									
PFOS	3.4	2.6	1.6–9.1	3.4	3.3	3.0–4.5	19.9	16.4	16–36
PFOA	7.8	7.6	6.6–10	11.2	10.2	6.6–24	9.4	8.8	8.5–11

**Alkylphenols and Bisphenol A (Alk-BPA)**									
Bisphenol A	443.0	450.9	400–470	326.9	354.7	170–385	1059	1162	756–1312
4-teroctylphenol	176.9	171.7	161–202	137.9	98.0	77–239	160.1	188.7	<LOQ-410
Nonylphenol	1492	1360	1304–1790	2006	1736	1187–3531	<LOQ	<LOQ	<LOQ
4-octylphenol	<LOQ	<LOQ	<LOQ	<LOQ	<LOQ	<LOQ	<LOQ	<LOQ	<LOQ

**Anthropogenic Biomarkers (AM)**									
Caffeine	92337	85747	75000–113000	49178	49965	43000–55000	31795	34192	22400–36000
1-methylxanthine	1346	1235	1100–1700	3748	3790	3200–4400	9445	9663	4800–16400
Paraxanthine	28395	29678	24000–32000	24565	23896	21400–31000	11939	11836	9450–14200
Nicotine	21568	21253	17000–26000	9245	8769	4300–13400	6855	5437	1500–11700
Cotinine	1522	1507	1450–1650	1818	1838	1600–2000	941	927	800–1100

**Table 4:** Means, medians and ranges of pharmaceuticals (*PHARM*) measured in effluent wastewater.

**Effluent WW**									
**Concentrations (ng/L)**	**WWTP A**			**WWTP B**			**WWTP C**		
	**Mean**	**Median**	**Range**	**Mean**	**Median**	**Range**	**Mean**	**Median**	**Range**
**Antibiotics**									
Amoxicillin	<LOQ	<LOQ	<LOQ	2.5	1.0	LOQ-17	45.5	42.0	35–62
Ciprofloxacin	141.0	137.5	47–246	172.4	176.5	112–248	293.7	285.4	205–389
Clarithromycin	281.6	254.4	101–516	312.7	317.8	216–395	802.6	830.9	740–847
Dehydro-erythromycin	176.3	162.5	44–345	148.5	157.8	85–221	280.1	282.5	231–327
Erythromycin	<LOQ	<LOQ	<LOQ	<LOQ	<LOQ	<LOQ	<LOQ	<LOQ	<LOQ
Lincomycin	10.2	9.2	5.9–15.8	17.6	16.1	13–24	20.9	21.9	17–26
Ofloxacin	215.1	203.2	83–380	275.4	267.3	190–360	390.2	393.2	254–542
Oxytetracycline	<LOQ	<LOQ	<LOQ	<LOQ	<LOQ	<LOQ	<LOQ	<LOQ	<LOQ
Spiramycin	<LOQ	<LOQ	<LOQ	73.4	64.0	LOQ-178	<LOQ	<LOQ	<LOQ
Sulfamethoxazole	78.8	67.2	17–382	70.7	58.7	LOQ-173	<LOQ	<LOQ	<LOQ
Vancomicin	37.0	31.5	LOQ-94	7.3	1.0	LOQ-24	<LOQ	<LOQ	<LOQ

**Anticancer**									
Cyclophosphamide	4.1	3.9	2.1–7.4	2.9	3.4	LOQ-5	2.5	3.6	LOQ-3.7
Methotrexate	<LOQ	<LOQ	<LOQ	<LOQ	<LOQ	<LOQ	<LOQ	<LOQ	<LOQ
Tamoxifen	<LOQ	<LOQ	<LOQ	<LOQ	<LOQ	<LOQ	<LOQ	<LOQ	<LOQ

**Antiinflammatory**									
Diclofenac	368.5	294.3	147–812	507.4	488.3	392–694	532.4	495.2	420–788
Ibuprofen	0.9	0.7	LOQ-2.8	8.1	0.7	LOQ-43	170.4	163.4	99–265
Ketoprofen	92.4	69.1	25–220	257.9	133.3	13–735	169.7	133.0	61–316
Naproxen	31.0	28.6	14–58	90.8	51.9	2–608	505.4	505.2	408–598
Paracetamol	<LOQ	<LOQ	<LOQ	<LOQ	<LOQ	<LOQ	22.6	19.8	17–36

**Bronchodilator**									
Salbutamol	5.4	5.2	2.8–11	10.2	9.5	7–19.1	6.9	6.7	5.1–9.0

**Cardiovascular**									
Atenolol	183.9	188.2	128–232	309.0	250.2	77–658	1083.6	1035.4	895–1362
Enalapril	<LOQ	<LOQ	<LOQ	<LOQ	<LOQ	<LOQ	12.0	11.1	7.4–18.4

**CNS drug**									
Carbamazepine	207.4	180.3	123–353	302.1	288.8	249–406	226.2	219.5	194–269
Demetyl-diazepam	3.9	3.9	1.8–7.3	6.0	5.8	4.3–8.1	4.0	3.6	3.2–6.2
Diazepam	1.4	1.4	0.8–2.2	2.0	1.8	1.3–3.3	5.5	4.4	2.1–16.1

**Diuretics**									
Furosemide	186.4	171.5	118–295	980.7	911.9	204–1852	494.8	500.2	443–563
Hydrochlorothiazide	442.5	302.2	11.9–2270	469.2	399.7	7–1074	136.6	127.3	17–276

**Estrogens**									
17-β estradiol	<LOQ	<LOQ	<LOQ	<LOQ	<LOQ	<LOQ	<LOQ	<LOQ	<LOQ
Estrone	<LOQ	<LOQ	<LOQ	2.8	2.2	LOQ-2.8	8.5	8.7	6.3–10.1
17-α ethynilestradiol	<LOQ	<LOQ	<LOQ	<LOQ	<LOQ	<LOQ	<LOQ	<LOQ	<LOQ

**Gastrointestinal**									
Omeprazole	<LOQ	<LOQ	<LOQ	<LOQ	<LOQ	<LOQ	<LOQ	<LOQ	<LOQ
Ranitidine	8.0	7.1	LOQ-15	78.2	79.1	47–124	148.4	154.4	87–174

**Lipid Regulators**									
Atorvastatine	1.9	1.3	LOQ-4	20.2	19.7	8.3–43.2	10.4	10.0	7.0–13.7
Bezafibrate	9.7	9.6	4.4–16.7	89.0	55.6	10.8–256	1271.6	1365.3	138–2700
Clofibric acid	<LOQ	<LOQ	<LOQ	0.4	0.2	LOQ-0.7	<LOQ	<LOQ	<LOQ
Gemfibrozil	4.7	3.3	2.4–9	20.7	9.4	1.2–60	43.0	41.8	31–64

**Erectile dysfunction drug**									
Sildenafil	<LOQ	<LOQ	<LOQ	<LOQ	<LOQ	<LOQ	<LOQ	<LOQ	<LOQ

**Table 5:** Means, medians and ranges of illicit drugs (*IDs*) measured in effluent wastewater.

**Effluent WW**									
**Concentrations (ng/L)**	**WWTP A**			**WWTP B**			**WWTP C**		
	**Mean**	**Median**	**Range**	**Mean**	**Median**	**Range**	**Mean**	**Median**	**Range**
**Cocaine and metabolites**									
Benzoylecgonine	11.3	10.4	7.0–22.0	5.8	0.9	<LOQ-40	126.8	116.7	100–170
Norbenzoylecgonine	4.1	3.7	3.0–7.0	8.4	6.5	3.0–25.0	8.2	7.6	6.0–11.0
Cocaine	0.9	0.8	0.5–1.3	0.5	0.3	<LOQ-2	26.8	24.3	22.0–36.0
Norcocaine	<LOQ	<LOQ	<LOQ	<LOQ	<LOQ	<LOQ	0.9	0.8	0.6–1.3
Cocaethylene	<LOQ	<LOQ	<LOQ	<LOQ	<LOQ	<LOQ	0.6	0.5	0.4–1.0
Ecgonine methyl ester	<LOQ	<LOQ	<LOQ	<LOQ	<LOQ	<LOQ	22.9	19.8	18.0–34.0
Ecgonine	<LOQ	<LOQ	<LOQ	<LOQ	<LOQ	<LOQ	31.9	20.4	<LOQ-48
Anhydroecgonine	7.8	7.2	3.0–13.0	25.0	24.6	16–38	16.1	17.9	12.0–19.0
Anhydroecg. methylester	<LOQ	<LOQ	<LOQ	<LOQ	<LOQ	<LOQ	<LOQ	<LOQ	<LOQ

**Opioids**									
Morphine	<LOQ	<LOQ	<LOQ	<LOQ	<LOQ	<LOQ	41.7	34.6	27–91
6-acethylmorphine	<LOQ	<LOQ	<LOQ	<LOQ	<LOQ	<LOQ	<LOQ	<LOQ	<LOQ
Morphine 3β glucuronide	<LOQ	<LOQ	<LOQ	<LOQ	<LOQ	<LOQ	<LOQ	<LOQ	<LOQ
Morphine 6β glucuronide	<LOQ	<LOQ	<LOQ	<LOQ	<LOQ	<LOQ	<LOQ	<LOQ	<LOQ
Oxycodone	<LOQ	<LOQ	<LOQ	<LOQ	<LOQ	<LOQ	<LOQ	<LOQ	<LOQ
Hydrocodone	<LOQ	<LOQ	<LOQ	<LOQ	<LOQ	<LOQ	<LOQ	<LOQ	<LOQ
Codeine	15.2	14.9	13.0–20.0	32.1	28.2	11.0–65.0	79.4	77.2	71–83
6-acethylcodeine	<LOQ	<LOQ	<LOQ	<LOQ	<LOQ	<LOQ	<LOQ	<LOQ	<LOQ
Methadone	8.8	8.8	7.0–11.0	14.9	15.5	10.0–21.0	8.4	8.5	7.0–10.0
EDDP	11.3	10.7	7.0–15.0	21.4	21.2	11.0–31.0	10.7	10.5	8.0–13.0

**Amphetamines and Ketamine**									
Amphetamine	<LOQ	<LOQ	<LOQ	<LOQ	<LOQ	<LOQ	<LOQ	<LOQ	<LOQ
Methamphetamine	24.9	24.0	14–37	27.3	18.3	1.5–79	4.0	3.6	3.0–6.5
MDA	<LOQ	<LOQ	<LOQ	<LOQ	<LOQ	<LOQ	<LOQ	<LOQ	<LOQ
MDMA	4.0	1.6	<LOQ-11	3.1	1.6	<LOQ-12	3.9	1.6	<LOQ-15
MDEA	<LOQ	<LOQ	<LOQ	<LOQ	<LOQ	<LOQ	<LOQ	<LOQ	<LOQ
MBDB	<LOQ	<LOQ	<LOQ	<LOQ	<LOQ	<LOQ	<LOQ	<LOQ	<LOQ
Ketamine	7.3	7.5	3.0–11.0	8.2	7.2	5.0–15.0	3.2	3.1	2.0–6.0

**Cannabinoids**									
THC-COOH	<LOQ	<LOQ	<LOQ	<LOQ	<LOQ	<LOQ	5.5	5.1	<LOQ-11
OH-THC	<LOQ	<LOQ	<LOQ	<LOQ	<LOQ	<LOQ	<LOQ	<LOQ	<LOQ
THC	<LOQ	<LOQ	<LOQ	<LOQ	<LOQ	<LOQ	<LOQ	<LOQ	<LOQ

**Table 6:** Means, medians and ranges of the other classes of ECs measured in effluent wastewater.

**Effluent WW**									
**Concentrations (ng/L)**	**WWTP A**			**WWTP B**			**WWTP C**		
	**Mean**	**Median**	**Range**	**Mean**	**Median**	**Range**	**Mean**	**Median**	**Range**
**Personal Care Products (PCPs)**									
PBSA	173.1	173.6	112–219	305.0	323.2	186–383	183.4	176.4	166–218
Benzophenone-4	185.9	179.6	141–283	406.9	419.6	231–750	133.8	132.9	112–155
Benzophenone-3	<LOQ	<LOQ	<LOQ	2.0	0.4	<LOQ-8	2.8	2.4	1.5–5.0
4-MBC	<LOQ	<LOQ	<LOQ	<LOQ	<LOQ	<LOQ	<LOQ	<LOQ	<LOQ

**Disinfectants (DIS)**									
Triclosan	<LOQ	<LOQ	<LOQ	150.7	173.2	<LOQ-244	329.7	312.0	287–390
Triclocarban	<LOQ	<LOQ	<LOQ	<LOQ	<LOQ	<LOQ	<LOQ	<LOQ	<LOQ

**Perfluorinated compounds (PERF)**									
PFOS	1.9	1.9	1.4–3.0	1.6	1.5	1.1–2.5	17.3	17.7	9.0–24.7
PFOA	12.2	12.4	9.5–15.0	14.3	13.85	9.0–20	10.1	10.05	9.8–10.5

**Alkylphenols and Bisphenol A (Alk-BPA)**									
Bisphenol A	2.5	1.9	<LOQ-5.0	24.5	24.1	16–35	51.0	47.1	36–70
4-teroctylphenol	3.6	1.35	<LOQ-14	1.7	1.35	<LOQ-4.0	<LOQ	<LOQ	<LOQ
Nonylphenol	183.5	180.9	144–264	73.0	72.1	10–197	<LOQ	<LOQ	<LOQ
4-octylphenol	<LOQ	<LOQ	<LOQ	<LOQ	<LOQ	<LOQ	<LOQ	<LOQ	<LOQ

**Anthropogenic Biomarkers (AM)**									
Caffeine	<LOQ	<LOQ	<LOQ	<LOQ	<LOQ	<LOQ	433	362	270–520
1-methylxanthine	<LOQ	<LOQ	<LOQ	<LOQ	<LOQ	<LOQ	<LOQ	<LOQ	<LOQ
Paraxanthine	<LOQ	<LOQ	<LOQ	<LOQ	<LOQ	<LOQ	120	123	71–170
Nicotine	229	204	114–365	69	79	<LOQ-110	454	461	288–725
Cotinine	13	12	11.0–14.0	11	11	10.0–13.0	126	126	110–140

**Table 7:** Concentrations of PHARM (ng/L) in surface water samples.

***PHARM***	***North of Milan***			***South of Milan***			
	***O1***	***S1***	***L1***	***L2***	***L3***	***L4***	***L5***
**Antibiotics**							
Amoxicillin	2.0	10.3	4.4	22.7	25.3	13.0	16.7
Ciprofloxacin	22.6	60.1	31.2	41.2	55.1	19.4	6.7
Clarithromycin	182	326	119	202	212	177	149
Dehydro-Erythromycin	61	94.7	30.2	73.3	60.6	58.0	53.2
Lincomycin	3.0	10.2	5.0	23.2	6.8	4.9	13.8
Ofloxacin	81.0	158	73.4	117	150	69.4	30.7
Sulfamethoxazole	3.2	1.3	6.3	1.6	9.5	13.9	10.1
Vancomicin	1.0	1.0	6.2	9.5	9.2	8.0	19.6

**Antiinflammatory**							
Diclofenac	86.5	184	60.0	695	461	215	121
Ibuprofen	76.5	134	53.5	174	107	62.6	79.5
Ketoprofen	3.9	9.8	30.8	26.8	20.1	8.5	0.9
Naproxen	52.4	92.7	71.1	124	122	75.7	62.4
Paracetamol	1.0	9.5	10.4	26.8	25.7	24.3	18.8

**Bronchodilator**							
Salbutamol	1.8	3.6	1.6	12.8	2.2	339	205

**Cardiovascular**							
Atenolol	110	400	171	280	232	184	166
Enalapril	1.5	6.5	2.6	7.1	5.7	4.4	3.6

**CNS drug**							
Carbamazepine	115	166	54.2	246	105	78.4	86.0
Diazepam	0.4	0.8	0.3	2.3	2.7	125	53
Demetyl-diazepam	0.8	1.7	0.7	1.6	1.1	66.3	38.0

**Diuretics**							
Furosemide	33.0	74.3	70.3	72.2	77.7	57.2	27.0
Hydrochlorothiazide	23.2	74.1	31.9	46.9	77.1	649	314

**Estrogens**							
17-β estradiol	1.3	4.0	2.3	3.2	2.5	2.5	1.3
Estrone	5.0	12.6	7.8	20.4	11.7	7.9	5.4

**Gastrointestinal**							
Ranitidine	7.0	14.4	4.2	10.6	8.5	4.0	5.1

**Lipid Regulators**							
Atorvastatine	1.4	4.2	1.6	3.3	2.3	0.8	0.8
Bezafibrate	12.3	22.2	9.9	21.3	148	79.5	28.2
Clofibric acid	5.4	1.4	0.2	41.2	0.2	0.2	8.4
Gemfibrozil	15.0	27.5	8.5	23.3	19.0	9.0	11.5

**Table 8:** Concentrations of *IDs* (ng/L) in surface water samples.

**IDs**	***North of Milan***			***South of Milan***			
	***O1***	***S1***	***L1***	***L2***	***L3***	***L4***	***L5***
**Cocaine and metabolites**							
Benzoylecgonine	33.5	74.8	38.5	82.1	65.4	39.2	45.4
Norbenzoylecgonine	2.9	7.5	3.7	6.6	4.9	3.2	3.1
Cocaine	3.9	21.2	10.1	33.3	18.9	12.2	12.0
Norcocaine	0.1	0.7	0.4	0.7	0.7	0.4	0.4
Cocaethylene	0.1	0.3	0.2	0.3	0.2	0.2	0.3
Ecgonine methyl ester	4.9	6.2	3.7	20.3	10.7	8.8	9.9
Anhydroecgonine	9.0	21.3	6.0	14.1	6.6	12.2	7.9

**Opioids**							
Morphine	0.3	1.6	2.5	2.0	6.2	8.2	1.5
Codeine	15.4	23.0	9.6	20.6	15.7	10.7	10.2
Methadone	2.5	9.7	1.8	8.0	3.7	2.6	2.7
EDDP	4.7	15.9	4.2	10.2	7.3	4.2	3.3

**Amphetamines and Ketamine**							
Methamphetamine	0.2	1.1	0.2	2.7	0.9	0.8	0.8
MDMA	0.2	0.2	1.2	1.5	1.5	1.3	0.5
Ketamine	40.8	4.1	0.6	3.4	1.4	1.0	1.8

**Cannabinoids**							
THC-COOH	0.7	1.4	2.0	3.5	2.7	1.9	2.1

**Table 9:** Concentrations of the others ECs (ng/L) in surface water samples.

	***North of Milan***			***South of Milan***			
	***O1***	***S1***	***L1***	***L2***	***L3***	***L4***	***L5***
**Personal Care Products (PCPs)**							
PBSA	167	517	105	294	174	167	124
Benzophenone-4	168	373	109	241	172	142	112
Benzophenone-3	4.1	13.7	3.8	9.1	6.6	4.7	2.8

**Disinfectants (DIS)**							
Triclosan	35.4	149	59.8	52.2	131	117	86.6

**Perfluorinated compounds (PERF)**							
PFOS	4.2	6.6	4.4	4.9	12.7	6.2	14.2
PFOA	25.1	33.8	13.1	26.5	16.7	11.7	18.4

**Alkylphenols and Bisphenol A (Alk-BPA)**							
Bisphenol A	90.1	295	126	154	119	131	114
4-ter-octylphenol	14.6	110	14.1	18.7	22.4	14.8	11.1
Nonylphenol	38.4	277	33.9	51.9	33.7	27.8	24.3

**Anthropogenic Biomarkers (AM)**							
Caffeine	885	4339	1519	3344	2903	2126	1644
1-methylxanthine	5.3	5.3	5.3	37.7	35.0	5.3	5.3
Paraxanthine	105	367	180	300	329	236	184
Nicotine	673	6424	2259	3334	3015	2033	1254
Cotinine	50.7	148	53.2	118	110	78.1	70.4

## 2 Experimental design, materials, and methods

### 2.1 Sample extraction and analysis

#### 2.1.1 Pharmaceuticals (PHARM) and illicit drugs (IDs)

PHARM and IDs were analysed updating methods already published [2], [3], [4]. Briefly, samples (50 mL of influent wastewater; 100 ml of effluent wastewater; 400 mL of surface water; 500 mL of groundwater) were acidified to pH 2.0 with 37% HCl, spiked with labeled internal standards and SPE-extracted using mixed reverse-phase cation exchange cartridges (Oasis MCX). Cartridges were conditioned before use by washing with 5 mL of methanol, 3 mL of ultrapure (MilliQ) water and 3 mL of water acidified to pH 2. Samples were passed through the cartridges at a flow rate of 5–15 mL/min depending on the volume. Cartridges were then vacuum-dried for 10 min and eluted with 2 mL of methanol and 2 mL of a 2% ammonia solution in methanol. The eluates were pooled, dried under a nitrogen stream and redissolved in ultrapure water (200 µL) for instrumental analysis.

Analyses were done using an API 3000 QqQ equipped with a Turbo Ion Spray source (AB- Sciex, Thornhill, Ontario, Canada), two Series 200 pumps and Series 200 auto-sampler (Perkin-Elmer, Norwalk, CT). Chromatographic separation was done using a Luna C8 50 mm×2 mm, 3 µm particle size (Phenomenex, Torrance, CA, USA) for PHARM and an XTerra MS C18, 100×2.1 mm, 3.5 µm (Waters Corp., Milford, MA) for IDs. Analytical conditions and validation parameters are described elsewhere [2], [3], [4].

Specific extraction and analytical conditions were adopted for a group of small polar metabolites of cocaine (called ecgonines) as detailed in an earlier publication [5]. The main differences were the volumes of extraction (20, 40 and 100 mL respectively for influent, effluent and surface water); the SPE cartridges (Oasis-MCX 150 mg); and sample reconstitution (eluates were dried to 20 µL and 80 µL of acetonitrile were added). In view of the high polarity of these substances, chromatographic separation was done with an XBridge HILIC 100×2.1 mm, 3.5 µm (Waters Corp., Milford, MA). Analytical conditions and validation parameters are described elsewhere [5].

#### 2.1.2 Personal care products, disinfectants, perfluorinated substances, alkylphenols and BPA

Specific analytical methods were developed and validated adapting already published methods for PCPs [6], DIS [7] and Alk-BPA [8]. A novel method was developed for PERF, described by Castiglioni et al., [9]. All these substances were extracted using the same SPE procedure. Samples (100, 200, 400 and 500 mL respectively for influent, effluent, surface and groundwater) were extracted using 3 mL HLB cartridges (60 mg Oasis HLB resin) and maintaining a neutral pH (7). Cartridges were conditioned by washing with 5 mL methanol and 3 mL Milli-Q water and samples were loaded at a constant flow rate from 5–15 mL/min depending on the volume. Cartridges were vacuum-dried and eluted with 4 mL methanol. Eluates were divided into two parts (2 mL each) for separate mass spectrometric analysis.

The first aliquot was used for PERF analysis and an API 3000 QqQ equipped with a Turbo Ion Spray source (AB- Sciex, Thornhill, Ontario, Canada) was used in the negative ionisation mode. Eluates evaporated to dryness under a nitrogen stream were reconstituted in 200 µL of methanol and Milli-Q water (40:60, v/v). Chromatographic separation was done using an XTerra MS C18, 100×2.1 mm, 3.5 µm column (Waters Corp., Milford, MA) as detailed elsewhere [9].

The second aliquot was used for PCPs, DIS, Alk-BPA analysis. A 6410 QqQ (Agilent Technologies, Santa Clara, CA, USA) was used in positive and negative ionisation mode, respectively for analysis of PCPs and DIS. Eluates were dried and reconstituted in 200 µL of MilliQ water. Chromatographic separation was carried out using an Atlantis T3 column 150×2.1 mm, 3 µm (Waters Corp., Milford, MA,USA). Analytical details and method validation are reported by [10]. The same extract was used for the analysis of Alk-BPA, with an API 3000 QqQ in negative ionisation mode as detailed elsewhere [8].

#### 2.1.3 Anthropogenic markers

The SPE method for the selected analytes was modified from previous publications for caffeine and nicotine analyses [11], [12] and included some of the main metabolites as described by Senta et al., [13]. The extraction volumes were 3, 200, 400 and 500 mL respectively for influent, effluent, surface and groundwater. Sample pH was adjusted to 7.0–7.5 using 12% HCl (v/v) and SPE was done with Oasis HLB cartridges previously equilibrated with 6 mL of methanol and 3 mL of ultrapure water. After loading the samples, cartridges were vacuum-dried for 5 minutes then eluted with 2 mL of methanol. Dried residues were redissolved in 100 μL of water/methanol mixture (80/20, v/v). Chromatographic separation was done using a 100×1 mm X-Terra C18 column (Waters Corp., Milford, MA,USA). Chromatographic and mass spectrometric conditions for analyses are described elsewhere [13].
